# Neuropathology in intrauterine growth restricted newborn piglets is associated with glial activation and proinflammatory status in the brain

**DOI:** 10.1186/s12974-018-1392-1

**Published:** 2019-01-08

**Authors:** Julie A. Wixey, Kah Meng Lee, Stephanie M. Miller, Kate Goasdoue, Paul B. Colditz, S. Tracey Bjorkman, Kirat K. Chand

**Affiliations:** 1 UQ Centre for Clinical Research, Faculty of Medicine, The University of Queensland, Herston, QLD 4029 Australia; 2 Institute of Health Biomedical Innovation (IHBI), Queensland University of Technology, Brisbane, Australia; 3 Perinatal Research Centre, Royal Brisbane and Women’s Hospital, Herston, QLD 4029 Australia

**Keywords:** White matter, Neurons, Microglia, Astrocytes, Neonatal brain injury

## Abstract

**Background:**

The fetal brain is particularly vulnerable to intrauterine growth restriction (IUGR) conditions evidenced by neuronal and white matter abnormalities and altered neurodevelopment in the IUGR infant. To further our understanding of neurodevelopment in the newborn IUGR brain, clinically relevant models of IUGR are required. This information is critical for the design and implementation of successful therapeutic interventions to reduce aberrant brain development in the IUGR newborn. We utilise the piglet as a model of IUGR as growth restriction occurs spontaneously in the pig as a result of placental insufficiency, making it a highly relevant model of human IUGR. The purpose of this study was to characterise neuropathology and neuroinflammation in the neonatal IUGR piglet brain.

**Methods:**

Newborn IUGR (< 5th centile) and normally grown (NG) piglets were euthanased on postnatal day 1 (P1; < 18 h) or P4. Immunohistochemistry was utilised to examine neuronal, white matter and inflammatory responses, and PCR for cytokine analysis in parietal cortex of IUGR and NG piglets.

**Results:**

The IUGR piglet brain displayed less NeuN-positive cells and reduced myelination at both P1 and P4 in the parietal cortex, indicating neuronal and white matter disruption. A concurrent decrease in Ki67-positive proliferative cells and increase in cell death (caspase-3) in the IUGR piglet brain was also apparent on P4. We observed significant increases in the number of both Iba-1-positive microglia and GFAP-positive astrocytes in the white matter in IUGR piglet brain on both P1 and P4 compared with NG piglets. These increases were associated with a change in activation state, as noted by altered glial morphology. This inflammatory state was further evident with increased expression levels of proinflammatory cytokines (interleukin-1β, tumour necrosis factor-α) and decreased levels of anti-inflammatory cytokines (interleukin-4 and -10) observed in the IUGR piglet brains.

**Conclusions:**

These findings suggest that the piglet model of IUGR displays the characteristic neuropathological outcomes of neuronal and white matter impairment similar to those reported in the IUGR human brain. The activated glial morphology and elevated proinflammatory cytokines is indicative of an inflammatory response that may be associated with neuronal damage and white matter disruption. These findings support the use of the piglet as a pre-clinical model for studying mechanisms of altered neurodevelopment in the IUGR newborn.

## Introduction

A large proportion of intrauterine growth-restricted (IUGR) infants exhibit adverse long-term neurological outcomes such as sensory, learning and attention difficulties, behavioural issues, school failure, psychiatric disorders, epilepsy and cerebral palsy [1–4]. Clinical imaging studies in IUGR infants demonstrate structural alterations and changes in grey and white matter volume [5–7]. Structural changes that persist at 1 year of age are associated with developmental disabilities [6, 8] that persist well into adulthood [9]. However, few studies have focused on mechanisms of brain damage in the IUGR neonate. This being pertinent as no therapeutic interventions are currently available to prevent or treat brain damage in the IUGR newborn. Determining the underlying mechanisms behind grey and white matter damage in the IUGR infant will help guide the development and choice of suitable therapies to protect and promote healthy brain development in the vulnerable IUGR brain.

Neuronal and white matter impairment have been demonstrated in several animal models of IUGR [10–13]. However, mechanisms of neurodevelopmental impairment in the IUGR neonate are complex and not well understood. A number of mechanisms proposed to be involved in mediating cellular damage in the IUGR brain include excitotoxicity, oxidative stress, necrotic and apoptotic neurodegeneration and neuroinflammation [14, 15]. Neuroinflammation is regarded as a key mechanism in several neurodegenerative disorders and preliminary studies suggest it may also mediate abnormal brain development in the IUGR neonate [16]. Activation of glial cells exacerbates neuroinflammation and brain injury. In the IUGR brain, evidence suggests that the major inflammatory mediators are activated microglia, reactive astrocytes and proinflammatory cytokines [16]. In the preterm rodent model of hypoxic-ischemic (HI), the sustained presence of activated microglia and proinflammatory cytokines in the brain following an acute HI event has been associated with ongoing white matter and neuronal damage [17], though few studies have examined the potential negative impact of neuroinflammation in IUGR animal models.

Of the limited studies investigating inflammation in the IUGR brain, varying results have been reported [16]. Postnatal examination in different IUGR models has shown increased numbers of microglia and astrogliosis [18–23] while others report little change [20, 23, 24]. Only one study to date has examined the cytokine response in the IUGR brain [25]. In the guinea pig hypoxemic IUGR model, inflammatory cytokines are up-regulated in the fetal brain at 64–65 days gestation (full gestation = ~ 65 days) and related to severity of brain injury as demonstrated by increased apoptosis and neuronal loss at this time point [25]. These studies were largely carried out in small animal models. Given the conflicting results, it is essential to examine whether this potential inflammatory state is associated with ongoing neuronal and white matter impairment in a more translatable model. This information will be critical for the design and implementation of successful therapeutic interventions to protect the IUGR newborn brain. Evidence from fetal sheep studies has led to clinical trials of in utero interventions to reduce IUGR [26, 27]. However, there are pros and cons with all animal models; i.e. varying degrees of brain maturation at birth. The piglet is regarded an appropriate animal to examine altered brain development arising from comprising perinatal events [28–30]. The newborn piglet is similar to the human newborn infant in size, development, circulation, metabolism and cerebral maturation. The piglet brain is gyrencephalic and has a similar grey to white matter ratio [31] as well as brain growth spurt in the perinatal period similar to the human [32]. Furthermore, growth restriction in the piglet occurs spontaneously obviating the need for surgical induction of growth retardation. The piglet model of IUGR mimics many of the human pathophysiological outcomes associated with IUGR including asymmetrical growth restriction with brain sparing [33]. Asymmetrical growth restriction is the most common form of growth restriction in humans and occurs in around 70–80% of all IUGR newborns [34]. Disruption to fetal growth occurs mainly in the third trimester for asymmetric growth restriction with fetal circulatory redistribution occurring; that is blood flow is selectively redirected to the brain away from other peripheral organs to the brain resulting in ‘brain sparing’. Inadequate fetal growth in pigs is caused by alterations associated with placental insufficiency which is the most common cause of IUGR in the human population [33]. Therefore, data obtained from the piglet model translates well to human IUGR pathology.

In the current study, we used the spontaneously growth-restricted piglet as a model of human IUGR to examine neuropathology and neuroinflammatory mediators at birth (P1) and at postnatal day 4 (P4). We hypothesised that inflammation would be prevalent in the IUGR piglet brain not only on P1 but also on P4 and would be associated with ongoing neuronal and white matter disruption.

## Materials and methods

### Animals and tissue preparation

Large White piglets were obtained from The University of Queensland Gatton Piggery. Approval for this study was granted by The University of Queensland Animal Ethics Committee (MED/UQCCR/132/16/RBWH) and was carried out in accordance with National Health and Medical Research Council (NHMRC) guidelines (Australia) and ARRIVE guidelines.

Term piglets were born spontaneously and collected on first day of life (postnatal day 1 (P1); < 18 h). IUGR piglets were defined by birth bodyweight (< 10th percentile) [35–37]. Litter-matched pairs were obtained from multiple sows (*n* = 11). The IUGR piglet model is well-established and characterised by our group and others [11, 33]. This model is caused by placental insufficiency [33]; the most common cause of IUGR in the human population. On either P1 (NG *n* = 6; IUGR *n* = 6) or P4 (NG *n* = 6; IUGR *n* = 6) (equal males and females in each group), piglets were euthanased via an intracardiac injection of sodium pentobarbital (650 mg/kg; Lethabarb, Virbac, Australia). The brain was immediately removed, weighed, hemisected and coronally sliced. The right hemisphere sections were immersion fixed in 4% paraformaldehyde as previously described [38]. The parietal cortex from the left hemisphere was snap frozen in liquid nitrogen and stored at − 80 °C for molecular studies.

### Immunohistochemistry

Brain sections from the right hemisphere were embedded in paraffin and coronally sectioned 6 μm apart at the level of the brain containing the parietal cortex (Pig stereotaxic map, A 5.5 mm; Felix 1999). Sections were dewaxed and rehydrated using standard protocol. The rehydrated slides underwent heat-induced epitope retrieval in 10 mM citrate buffer (pH 6) at 80  C for 20 min before cooling to room temperature (RT).

Tissue sections were blocked with 5% donkey serum in phosphate-buffered saline (PBS) with 0.5% Triton-X 100 for 1 h at RT. Immunohistochemistry was performed on brain sections for visualisation of microglia (ionised calcium-binding adaptor molecule-1; Iba-1; 1:1000, ab5076; Abcam, Queensland, Australia), astrocytes (glial fibrillary astrocytic protein; GFAP; 1:1000, Z0334, Dako), neurons (NeuN; 1:1000, ab177487; Abcam), proliferating cells (Ki67; 1:200, ab15580; Abcam) and apoptotic cells (cleaved Caspase-3; 1:500, #9661; Cell Signalling). Co-localisation studies were also conducted for the inflammatory cytokines IL-1β (1:100, ab9722; Abcam), TNF-α (1:200, AF-410; R&D Systems, Minneapolis, MN), IL-18 (1:100, ab71495; Abcam), IL-4 (1:100, ab9622; Abcam) and nuclear factor-kappa beta (NF-kB p65: 1:500, ab16502; Abcam). Primary antibodies were incubated at 4 °C for 20 h. Slides were washed in tris-buffered saline followed by incubation with species-specific secondary fluorophores at RT for 1 h (Alexafluor 488, Alexafluor 594; 1:1000, Molecular Probes, Invitrogen Australia, Victoria, Australia). Tissue was then washed, counterstained with 4′,6-diamidino-2-phenylindole (DAPI) and mounted with Prolong Gold antifade (Molecular Probes, Invitrogen Australia, Victoria, Australia). Negative control sections without primary antibodies were processed in parallel. All staining was conducted in triplicate for each animal at each time point.

#### Luxol fast blue and Fluoro Jade C staining

We assessed the general myelination status of IUGR brains at P1 and P4 using Luxol fast blue (LFB) staining. Tissue sections underwent standard dewaxing and rehydration followed by overnight immersion in LFB solution at 57 °C. Sections were then immersed in 95% ethanol and differentiated in 0.05% lithium carbonate followed by 70% ethanol until grey and white matter could be distinguished and nuclei decolourized. Tissue was processed and stained simultaneously to minimise variability of LFB staining.

To investigate the number of degenerating neurons, Fluoro Jade C (FJC) was used. This is a dye that specifically stains all degenerating neurons [39]. Dewaxed and rehydrated slides were incubated in 0.006% potassium permanganate solution for 5 min. Slides were rinsed in PBS for 2 min then transferred to a 0.0001% solution of FJC (Merck Millipore, Germany) dissolved in 0.1% acetic acid vehicle, containing DAPI to counterstain the nuclei for 15 min. Slides were washed 2 × 1 min in PBS followed by air drying on a slide warmer at 50 °C for 5 min. Slides were cleared with xylene for 1 min before being coverslipped with DPX mounting media (Sigma-Aldrich).

### Image analysis

Analysis of immunolabelled sections were performed using an Olympus BX41 light microscope with a DP70 camera as previously described [40]. Photomicrographs (881.2 μm × 663.5 μm) of grey matter (parietal cortex) and white matter (intragyral white matter-IGWM; subcortical white matter-SCWM; periventricular white matter-PVWM) regions were captured for analysis. Four pictomicrographs were captured in each respective area in three sequential sections (50 μm apart) for each animal.

All tissue was imaged and analysed under blind conditions by KKC and JAW and manual counts for NeuN, FJC, Caspase-3, Ki67 and Iba-1 were performed. For LFB staining, slides were scanned using a Leica SCN400 Slide Scanner with a × 20 objective and analysed as previously reported [41]. Briefly, the WM was automatically outlined using the wand tool in ImageJ software; FIJI (NIH Bethesda, USA). Incidental border regions, such as large blood vessels, were excluded from the analysis. Scanned images were converted to greyscale to determine the staining intensity from 0 to 127 (0, white; 255, black). This range was divided into quartiles and the percent area (% area) calculated for each quartile. The median grey level of each quartile (14.5, 46.0, 78.5 and 111.0) was then multiplied by % area/100 in each quartile, to give the total myelin index. Therefore, the ratio is defined as myelination index (%).

GFAP-positive astrocytes in the WMTs were quantified using densitometry by thresholding the intensity of GFAP labelling using ImageJ (Image Processing and Analysis in Java; National Institutes of Health, Bethesda, MD, USA). Areal density was expressed as a percentage of the whole white matter for each region covered.

### Quantitative polymerase chain reaction

The Pig Inflammatory Cytokines & Receptors RT^2^ Profiler™ PCR Array (Qiagen, Hilden, Germany) profiles the expression of 84 key genes mediating the inflammatory response. Total RNA was purified using the RNeasy Tissue Mini Kit (Qiagen) from 30 mg of the parietal cortex. Total RNA concentration and quality was determined via UV spectroscopy using a NanoDrop (ND-1000 system). cDNA was synthesised from the purified total RNA via reverse transcription using the RT^2^ First Strand Kit (Qiagen). Synthesised cDNA were pooled for each group (total four groups: P1 NG *n* = 6, IUGR *n* = 6; P4 NG *n* = 6, IUGR *n* = 6), giving equal cDNA concentrations from each animal in the pooled sample. Pooled cDNA was added to the RT^2^SYBR Green Mastermix and pipetted into the Pig Inflammatory Cytokines & Receptors RT^2^ Profiler™ PCR Array. Polymerase chain reaction (PCR) was performed using a Rotor-Gene Q real-time cycler (Qiagen). The amplified transcripts were quantified with the comparative CT method using Glyceraldehyde 3-phosphate dehydrogenase (GAPDH) mRNA expression levels for normalisation. The same CT threshold value was used across all arrays to allow accurate comparison between runs.

### Statistical analysis

Two-way ANOVA with the post-hoc Sidak analysis was used to determine differences between NG and IUGR animals at each age (Graph Pad Prism 5.0 software, San Diego, California, USA). Results were expressed as mean ± SEM with statistical significance accepted at *p* < 0.05.

## Results

Body weight was significantly reduced in the IUGR piglets in comparison to NG at P1 (*p* = 0.0004) and P4 (*p* < 0.0001) (Table 1). Brain to body weight ratio was also significantly different between IUGR and NG at both P1 (*p* < 0.0001) and P4 (*p* < 0.0001) indicating asymmetric growth restriction in the IUGR piglets.

**Table 1:** Piglet bodyweight and brain weight

Piglet groups	P1		P4	
	IUGR (*n* = 6)	NG (*n* = 6)	IUGR (*n* = 6)	NG (*n* = 6)
Bodyweight in kilogrammes (mean ± SEM)	0.873 ± 47.16	1.507 ± 111.7***	0.770 ± 41.57	1.973 ± 157.4****
Brain weight in grams (mean ± SEM)	27.83 ± 1.142	30.48 ± 0.8878	27 ± 0.8375	32.19 ± 1.102**
Brain:body weight (g/kg)	32.1 ± 1.302	20.63 ± 1.246****	35.35 ± 1.263	16.63 ± 0.860****

Piglet bodyweight and brain weight. IUGR piglets had significantly lower mean bodyweight when compared to NG piglets in both age groups. Significantly lower mean brain weight was observed only at P4 in IUGR piglets compared to NG. IUGR piglets in both age groups demonstrated significantly higher brain to bodyweight ratio in comparison to respective NG groups. Values are the mean ± SEM. **p < 0.01; ***p < 0.001; ****p < 0.0001 IUGR versus NG

### Neuronal disruption in the IUGR piglet brain

**Fig. 1:**
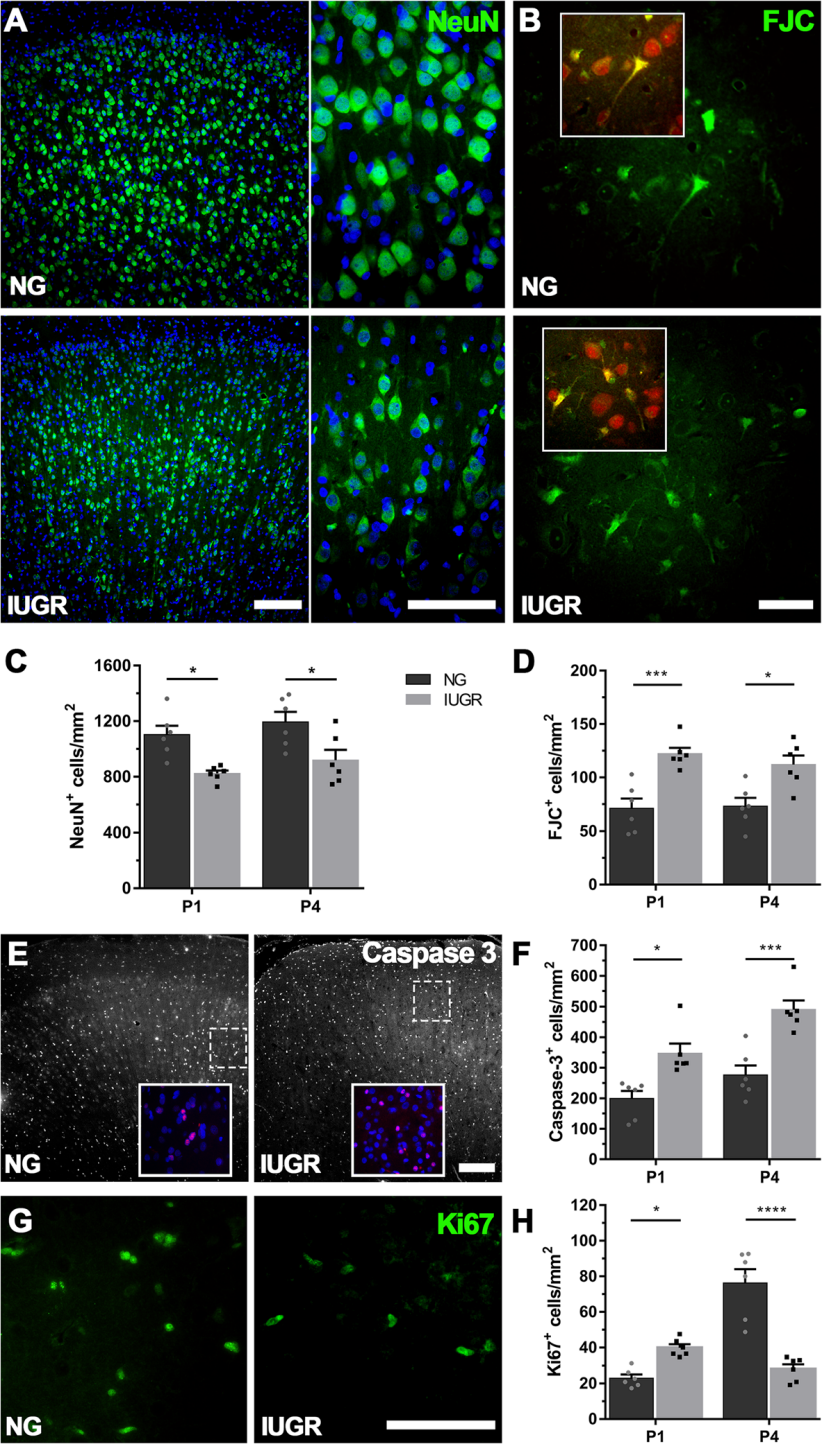
Altered expression of neurodevelopmental markers in the parietal cortex of IUGR newborn piglets. **a** Immunofluorescent staining of mature neurons using the neuron-specific nuclear marker NeuN in NG and IUGR brains at P4. NG brains displayed robust NeuN expression throughout the cortex, while IUGR consistently showed lower levels of expression. Scale bars = 100 μm. Quantification of expression found a decreased number of NeuN-positive labelled cells in IUGR at both P1 and P4 compared with age-matched NG brains (**c**). **b** Representative staining of degenerating neurons (Fluoro-Jade C positive cells, green) observed in both NG and IUGR brains at P4 (scale bar = 50 μm). FJC-positive cells displayed distinct staining of cell bodies and processes. These cells co-localised with neurons displaying lower expression of NeuN (see insets; NeuN-positive cells, red). **d** IUGR brains displayed significantly higher numbers of FJC-positive cells at both P1 and P4 when compared with NG. **e** Representative image of apoptotic cells (caspase 3-postive, magenta) in the cortex of both NG and IUGR at P1 and P4 (scale bar = 200 μm; DAPI, blue). **f** IUGR displayed higher apoptotic cell counts at both ages investigated. **g** Cellular proliferation was observed using Ki67 (scale bar = 50 μm). IUGR displayed higher expression at P1, while there was an observed increase in Ki67-postive cells in P4 NG brains (**h**). For **a** IUGR (P1 and P4 *n* = 6) and NG (P1 *n* = 5; P4 *n* = 6). Values are presented as mean ± SEM. Two-way ANOVA with Sidak post-hoc test, significance between IUGR and age-matched NG *p < 0.05; **p < 0.005; ***p < 0.001; ****p < 0.0001

Using immunohistochemistry, we examined the expression of healthy mature neurons labelled with NeuN, a neuronal nuclei marker. We demonstrated a 25.4% and 23.0% reduction in NeuN-positive cells in the IUGR parietal cortex in comparison with NG on P1 (*p* = 0.0267) and P4 (*p* = 0.0307) respectively (Fig. 1a, c). Qualitatively, we observed alterations in neuronal morphology as well as in the expression patterns of NeuN staining in the IUGR brain. NeuN-positive cells appeared to be smaller in size and displayed a hollow staining pattern, suggestive of a loss in cell nucleus expression, when compared with NG controls (Fig. 1a). We have previously shown impaired neuronal morphology in the IUGR piglet brain [11]; however, it was not clear whether the neurons were degenerating and undergoing cell death. Therefore, in the current study, we also examined neuronal degeneration using FJC. There was significant co-localisation of FJC with NeuN-positive cells (Fig. 1b), with these double-labelled cells displaying a more dense/shrunken morphology when compared with only NeuN-positive cells. We demonstrated a significant increase in FJC-positive cells in the IUGR parietal cortex on both P1 (41.3%, *p* = 0.0009) and P4 (34.7, *p* = 0.0114) in comparison to NG (Fig. 1b, d). To determine whether apoptotic cell death was concurrently occurring in the IUGR brains, we used the apoptotic marker cleaved caspase-3. We found the number of caspase-3-positive cells were significantly increased in IUGR parietal cortex at both P1 (42.7%, *p* = 0.0104) and P4 (43.8%, *p* = 0.0003) (Fig. 1e, f) compared with NG piglets indicating a state of apoptotic cell death in the IUGR brain. Ki67 immunolabelling was used to determine the number of proliferating cells in the parietal cortex. At P1, there was a significant increase in Ki67-positive cells in IUGR parietal cortex in comparison to NG (42.4%, *p* = 0.0267) (Fig. 1g, h). However, at P4, a reversal of this pattern occurred with a marked and significant decrease in Ki67-positive cells in the IUGR parietal cortex when compared with NG (63.2%, *p* < 0.0001).

### White matter disruption in the IUGR piglet brain

**Fig. 2:**
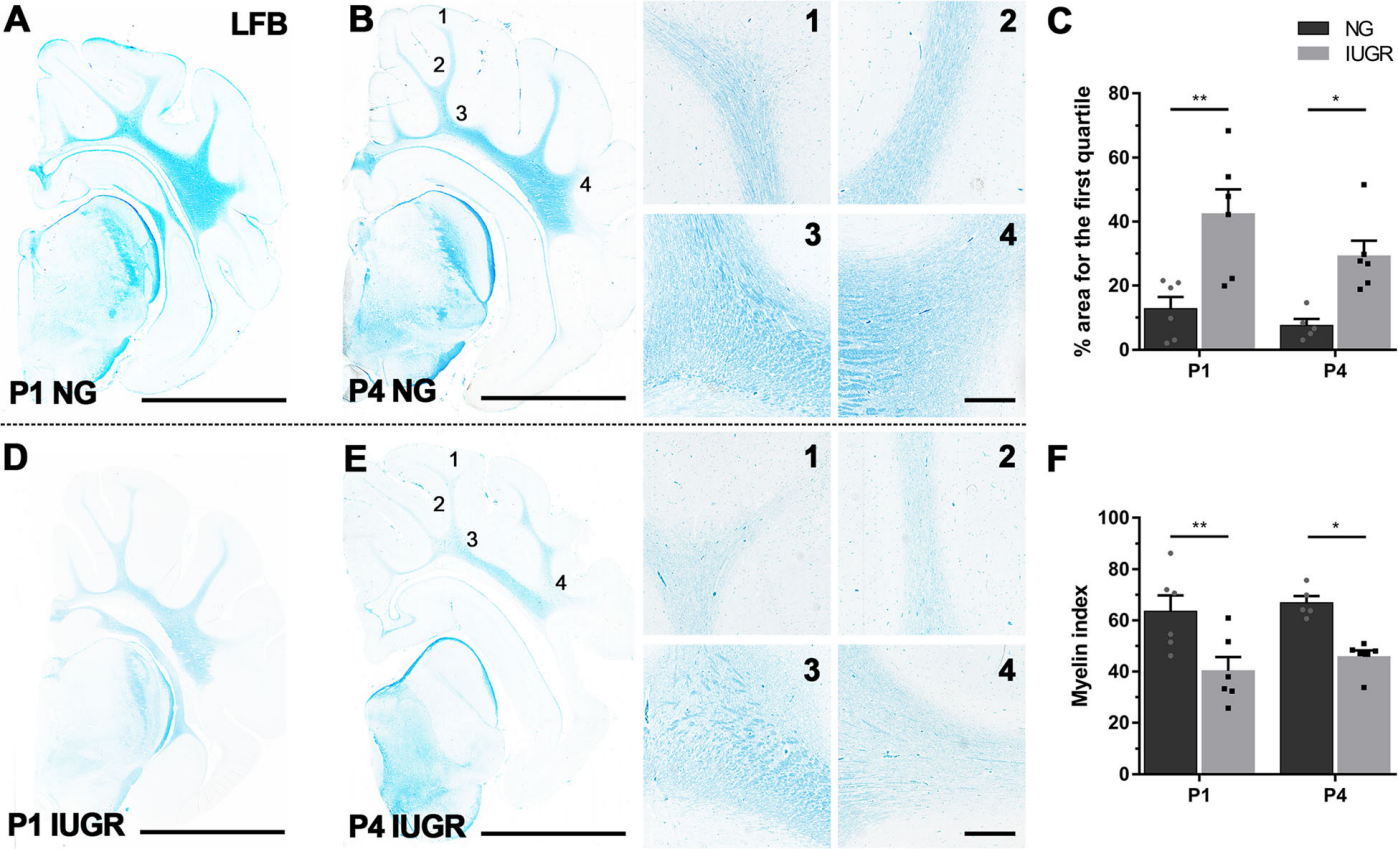
Decreased myelination status in white matter of IUGR newborn piglets. **a**, **b** Representative images demonstrating high expression of myelin stained with Luxol fast blue (LFB) in P1 and P4 NG compared with IUGR (**d**, **e**) brains (scale bar = 10 mm). The degree of LFB staining was consistent across all white matter (WM) regions in NG brains (**b(***1*–*4*)), while IUGR displayed overall lower levels with variability between WM regions (**e(***1*–*4*); scale bar = 500 mm). **c** IUGR brains presented greater areas with significantly lower LFB staining, as indicated by percentage area for the first quartile. **f** IUGR displayed a decreased myelin status as shown by the myelin index at both P1 and P4 in IUGR. For **c**, **f** IUGR (P1 and P4 *n* = 6) and NG (P1 *n* = 5; P4 *n* = 6). Values are presented as mean ± SEM. Two-way ANOVA with Sidak post-hoc test, significance between IUGR and age-matched NG *p < 0.05; **p < 0.005

Luxol fast blue (LFB) staining was used to observe myelination in the IGWM of the parietal lobe as well as deeper white matter regions including SCWM and PVWM. In NG brains, LFB staining revealed dense, well-organised white matter fibres, typical of the myelination pattern for a developing brain (Fig. 2b(1–4)). Yet in the IUGR brain, the myelination pattern appeared to be associated with a loss of axonal fibres (Fig. 2e(1–4)). Results reported are for all regions combined. The IUGR brains had significantly decreased myelination staining (i.e. less LFB staining), as indicated by the higher percentage for the first quartile at both P1 (*p* = 0.0030) and P4 (*p* = 0.0430) (Fig. 2c). Furthermore, decreased myelination status was evident by the myelin index at both P1 (*p* = 0.0094) and P4 (*p* = 0.0271) in IUGR brains in comparison to age matched NG (Fig. 2f). These data confirm our previous findings in the IUGR piglet of decreased white matter staining suggesting impaired myelination in the IUGR brain [11].

### Glial cell disruption and inflammation in the IUGR piglet brain

**Fig. 3:**
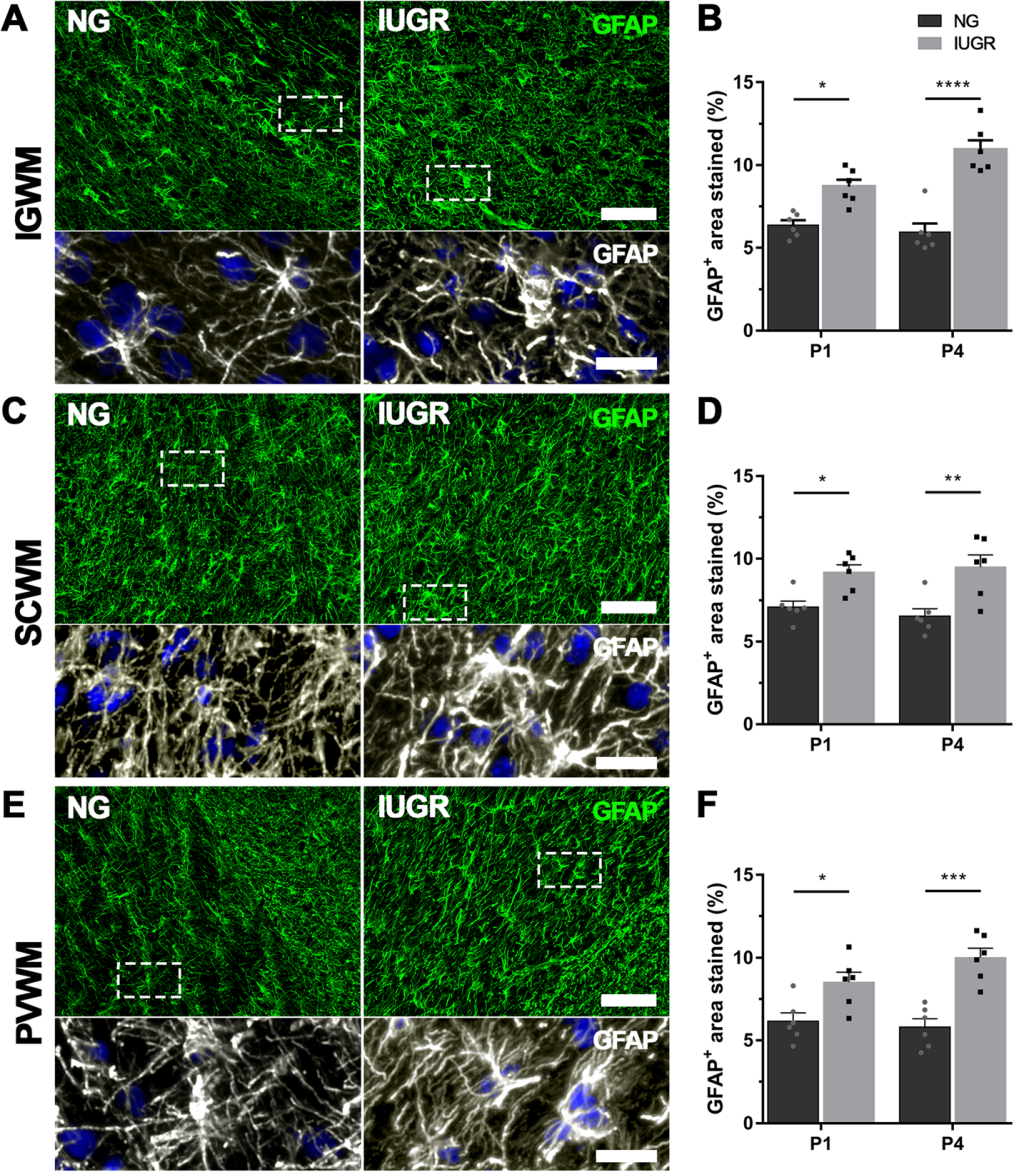
Increased astrogliosis in white matter of IUGR newborn piglets. **a** Representative images of astrocytes (GFAP) in the intragyral (**a**), subcortical (**c**) and periventricular (**e**) white matter regions in P4 NG and IUGR brains. Astrocytes in NG displayed long extended processes and small cell bodies, typical of ramified astrocytes (see NG bottom panels). Astrocytes in IUGR white matter displayed morphology characteristic of glia in activated states, with enlarged cell bodies and thickened processes (see IUGR bottom panels). Scale bar is 100 μm in low magnification images (top panels) and 20 μm in high magnification images (bottom panels). **b**, **d**, **f** Quantification of GFAP expression using densitometry showed an increase in GFAP expression in all white matter regions investigated at both P1 and P4 in IUGR when compared with age-matched NG brains. For **b**, **d**, **f** IUGR (P1 and P4 *n* = 6) and NG (P1 and P4 *n* = 6). Values are presented as mean ± SEM. Two-way ANOVA with Sidak post-hoc test, significance between IUGR and age-matched NG *p < 0.05; **p < 0.005; ***p < 0.001; ****p < 0.0001. *IGWM* intragyral white matter, *SCWM* subcortical white matter, *PVWM* periventricular white matter

In the NG brains, GFAP-positive astrocytes in the white matter tracts (WM) of the parietal region demonstrated multiple long branching processes from the cell body typical of normal astrocyte morphology. In the IUGR brains, many GFAP-positive astrocytes displayed morphology suggestive of a reactive state with larger cell bodies and fewer, retracted processes (Fig. 3). GFAP-positive astrocyte density was significantly increased in the IUGR brain in IGWM (*p* = 0.0111), SCWM (*p* = 0.0456) and PVWM (*p* = 0.0327) compared with NG brains on P1 (Fig. 3b, d, f). On P4, GFAP-positive astrocyte density remained significantly increased in the IUGR brain in all white matter regions, IGWM (*p* < 0.0001), SCWM (*p* = 0.0034) and PVWM (*p* = 0.0002) (Fig. 3b, d, f).

**Fig. 4:**
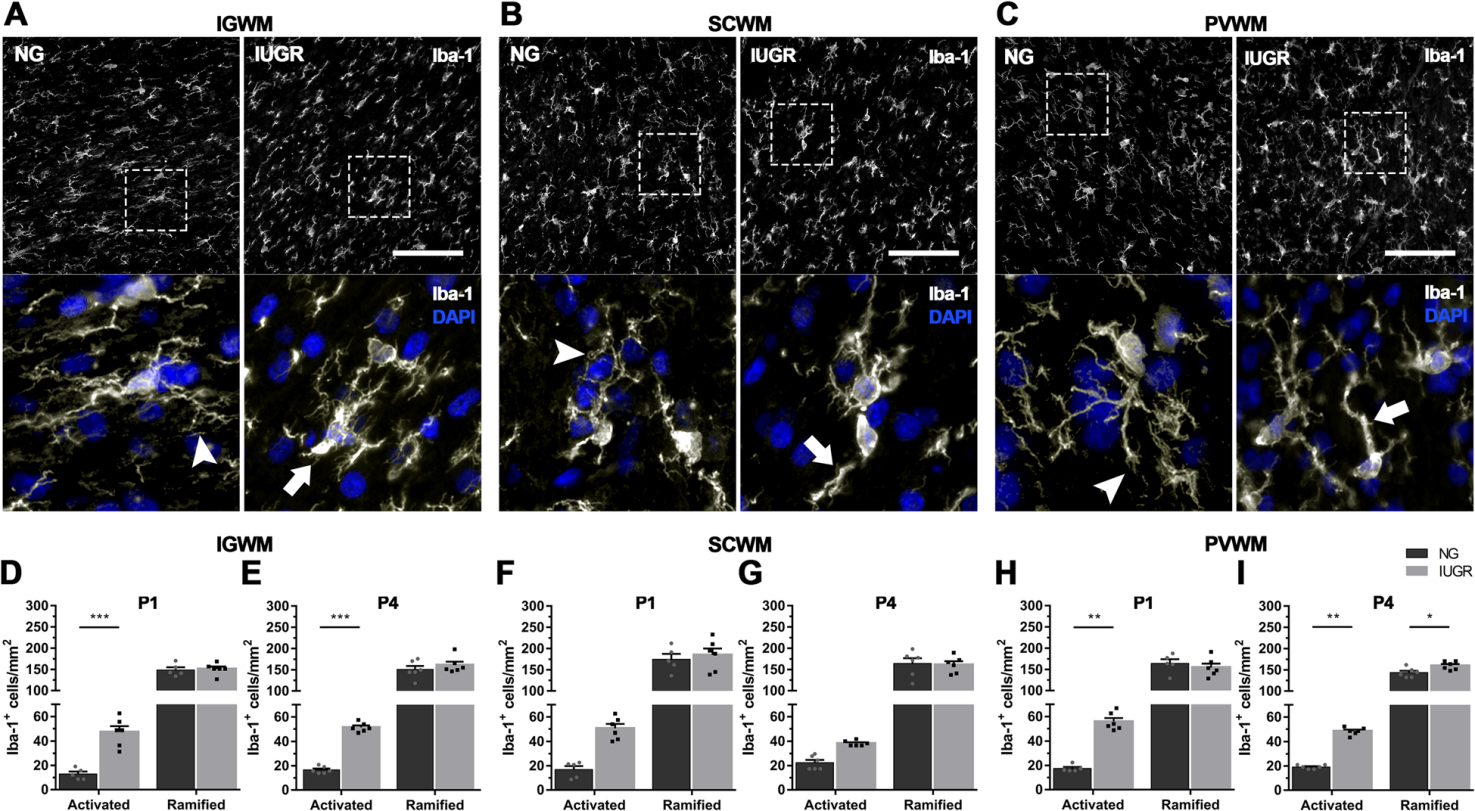
Increased microglial activation in WM of IUGR newborns. **a**–**c** representative images of microglial expression in the intragyral white matter (IGWM), subcortical white matter (SCWM) and the periventricular white matter (PVWM) of P4 control and IUGR brains. **d**, **e** Increased microglial activation was noted in the IGWM of IUGR brains at both P1 and P4, with no significant difference in the number of ramified cells based on morphological analysis. **f**, **g** No significant difference in microglial morphology was noted in the SCWM in IUGR compared with control at P1 and P4. **h**, **i** Increased microglial activation was noted in the PVWM of IUGR brains at both P1 and P4. At P4, there was also an increase in the number of ramified microglia in IUGR brains compared with control. For **d**–**i** IUGR (P1 *n* = 6; P4 *n* = 6) and control (P1 *n* = 6; P4 *n* = 6). Values are presented as mean ± SEM. Two-way ANOVA with Sidak post-hoc test, significance between IUGR and age-matched controls *p < 0.05; **p < 0.005

Number of microglia in the WM tracts of the parietal region were determined by Iba-1-positive cell counts. Ramified/resting microglia were characterised by light round or oval cell bodies with fine symmetrical extended processes, and activated microglia by darker cell bodies and thickened retracted processes (Fig. 4a–c). Iba-1-positive cells in the white matter regions of NG and IUGR examined typically possessed primary processes oriented in the direction of the axon bundles, with the finer processes orthogonally oriented. Significant increases in numbers of Iba-1-positive ramified microglia were only observed between IUGR and NG in the PVWM at P4 (*p* = 0.0136; Fig. 4i).

Significant increases in Iba-1-positive activated microglia were evident in the IUGR brain both on P1 and P4 in the IGWM (P1, *p* = 0.0003; P4, *p* = 0.0009; Fig. 4d, e), and PVWM (P1, *p* = 0.0017; P4, *p* = 0.0011; Fig. 4h, i) when compared to NG. No significant differences in Iba-1-positive activated microglia were evident in SCWM between IUGR and NG for either time point (Fig. 4f, g); however, a trend towards an increase in numbers was apparent in the IUGR brain at P1 and P4.

**Fig. 5:**
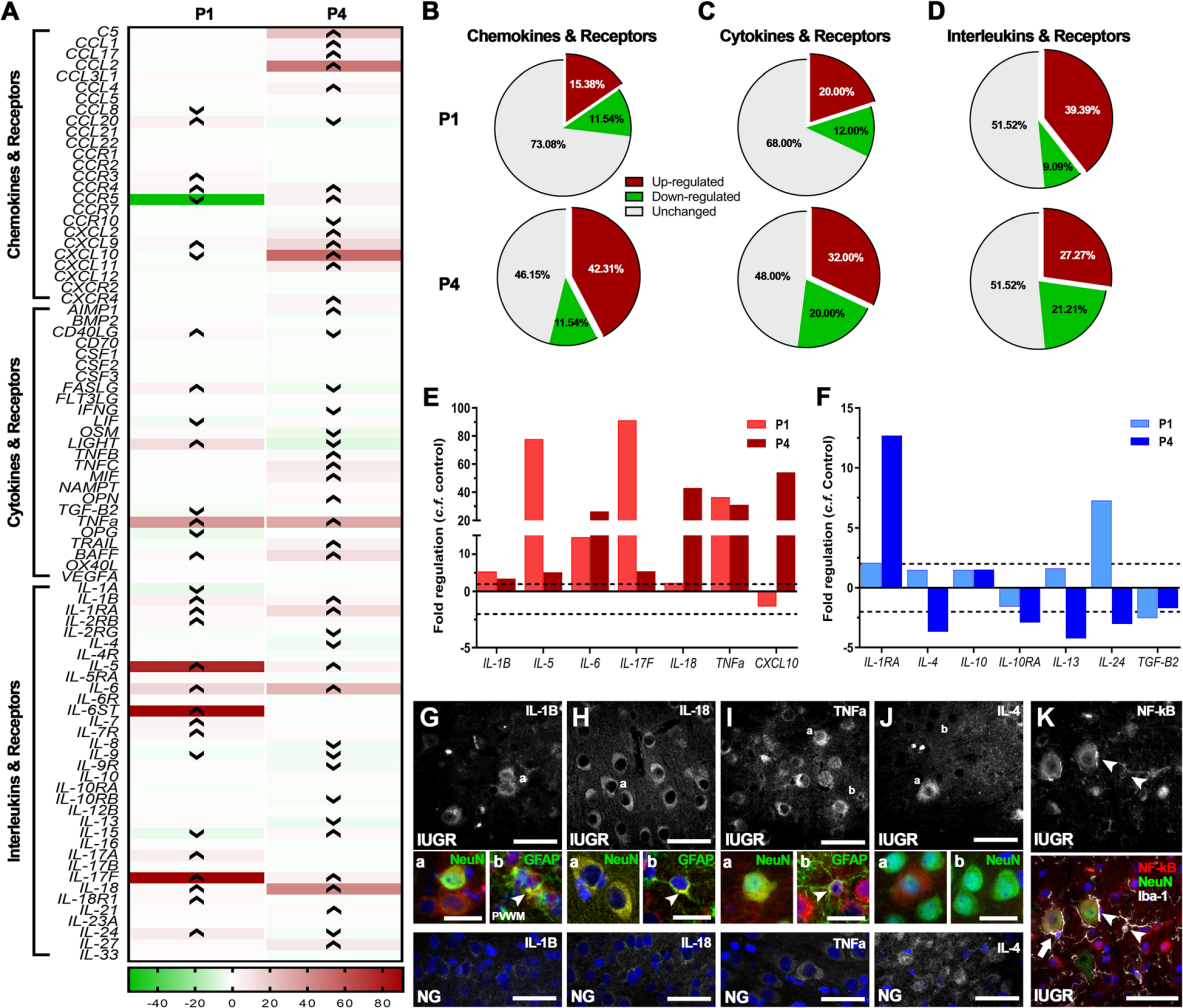
Altered expression of inflammatory mediators at P1 and P4 in IUGR newborn brains. **a** Heat map of inflammatory profiler array at P1 (first column) and P4 (second column) demonstrating altered expression of pro- and anti-inflammatory mediators in the cortex relative to age-matched controls. IUGR demonstrated an increase in the number of cytokines (**b**) and chemokines (**c**) that are up-regulated from P1 to P4. Interleukins and receptors displayed a general down-regulation of expression levels from P1 to P4 in IUGR brains. Expression of well-characterised proinflammatory (**e**) and anti-inflammatory (**f**) mediators at P1 and P4 relative to age-matched controls. IUGR demonstrated high expression of IL-1β (**g**), IL-18 (**h**) and TNF-α (**i**), which was co-localised to neurons (NeuN) and astrocytes (GFAP; arrow heads in **b** of **g**, **h**). Low expression of these proinflammatory mediators was noted in NG brains. **j** Low expression of IL-4 was observed in IUGR brains, in contrast to the high neuronal expression observed in NG. **k** IUGR displayed NF-kB staining in neurons and microglia as indicated by arrow heads. High expression was observed in microglial processes that were in close proximity to neurons expressing NF-kB (arrow). For (**g**–**k**) scale bar = 50 μm, for high magnification images (**a**, **b**) scale bar = 20 μm

Using a PCR array panel of 84 inflammatory genes, we observed altered expression of both pro- and anti-inflammatory cytokines in the IUGR parietal cortex relative to NG at both P1 and P4 (Fig. 5a). There was a marked increase of chemokine and cytokine mRNAs displaying up-regulated expression in IUGR brains at P4 when compared with P1(Fig. 5b, c). There was an increase in the percentage of down-regulated interleukin mRNAs in IUGR animals at P4 when compared with P1 (Fig. 5d). These findings show the dynamic neuroinflammatory alterations in response to IUGR and indicate an ongoing age-associated inflammatory state which may be driven by elevated expression of chemokines and cytokines. The proinflammatory mediators IL-1β, IL-6 and TNF-α showed high up-regulation in IUGR at P1 when compared with NG (Fig. 5e). By P4, there appeared to be some modulation in the degree of up-regulation; however, higher expression of these common markers was maintained compared with NG. Anti-inflammatory mediators IL-4, IL10 and TGF-β2 tended towards a decrease in expression both at P1 and P4 (Fig. 5f).

To identify cell types that expressed these pro- and anti-inflammatory markers, we performed double immunohistochemistry using cell-specific markers: NeuN (neurons), GFAP (astrocytes) and Iba-1 (microglia). Our qualitative observations found that proinflammatory cytokines IL-1β, IL-18 and TNF-α (Fig. 5g, h, i) were primarily co-localised to neurons in the IUGR parietal cortex with fewer cells and lower expression noted in NG piglets. GFAP-positive astrocytes co-localised with IL-1β were only observed in the PVWM (Fig. 5g, b), which was in contrast to astrocytes expressing IL-18 and TNF-α that were observed in the parietal cortex (Fig. 5b, h, i). Low labelling of the anti-inflammatory cytokine IL-4 was observed in IUGR brains, in contrast to the high expression in NG (Fig. 5j). We also observed high expression of NF-κB p65 which is induced by TNF-α and IL-1β to initiate an inflammatory response. NF-κB p65 was found to co-localise with neurons and microglia in P4 IUGR cortex (Fig. 5k). Qualitatively, we observed close interaction between NF-κB-positive microglia and NF-κB-positive neurons, with microglial processes appearing to wrap around the neuronal cells (Fig. 5k).

## Discussion

The current study demonstrates neurodevelopmental abnormalities of both grey and white matter which may be associated with the observed inflammatory response in the newborn IUGR piglet brain. Neuronal degeneration and cell death were evident in the parietal lobe of the P1 and P4 IUGR piglet brain. The persistent inflammatory state as noted by activated microglia, astrocytes and proinflammatory cytokines may be responsible for ongoing or exacerbation of neuronal and white matter disruption in the IUGR brain.

### Neuronal disruption in the IUGR piglet brain

At the macroscopic level, significant alterations in brain volume and structure are observed in the human population [5–7]. Cortical grey matter volume in IUGR infants is 28% less than that of age-equivalent healthy term-born infants [5]. While disturbances to the cortical grey matter in the IUGR infant are noted in the human population at a macroscopic level, evidence of alterations in neuronal architecture at the immunohistochemical level has been drawn from IUGR animal studies, with altered cortical neuronal morphology reported in the fetal sheep and piglet brain [10, 11]. In the current study, we observed decreased neuronal number that was associated with neuronal degeneration and in some instances cell death suggesting impairment of neuronal development rather than a delay in neuronal maturation in the IUGR brain. Both neuronal degeneration and apoptotic cell death were evident at P1 and P4 in the IUGR piglet brain which may be indicative of ongoing impairment that extends beyond that which occurred during fetal life. An increase in apoptotic cells have also been reported in both the IUGR fetal sheep and postnatal rat [10, 18]. Furthermore, multiple IUGR animal studies have demonstrated decreased numbers of neuronal cell counts in IUGR brains [12, 13, 25, 42]. In contrast to the current study, no degenerating neurons were observed in the IUGR fetal sheep [10]. This could be due to the time point of brain analysis (fetal vs postnatal) or the method of detection. Alves de Alencar Rocha et al. used morphological identification of ‘unhealthy’ NeuN-positive cells rather than direct measurement of degenerating neurons with FJC staining. Further to this, while noting disrupted neuronal morphology, these authors did not observe a reduction in total neuronal cell number in the fetal sheep brain; although they report increased apoptosis through cleaved caspase-3 labelling in the late-onset growth restriction model.

We found no co-localisation of Ki67-positive proliferating cells with NeuN-positive cells in our piglet brains (data not shown); it is possible that some of these proliferating cells are immature neurons attempting to regenerate. This theory is supported by a study in an IUGR guinea pig model demonstrating Ki67-positive cells in subventricular zone of the IUGR fetal brain were predominantly immature neurons [24]. They also reported increased Ki67-positive cells in the IUGR fetal brain similar to our findings on day of birth in the parietal cortex. The impaired newborn brain may be subject to not only altered cellular proliferation but also degeneration of immature neurons. Whether the increase in proliferating cells we observed in the current study at P1 are immature neurons remains to be determined. In contrast by P4, we demonstrated a decrease in proliferating cells. A reduction in Ki67-positive proliferative cells has also been reported in an IUGR rat model postnatally at both P3 and P10 [18]. Whether this decrease in proliferation persists long-term and what effect it has on neurodevelopmental outcomes remains to be determined.

The parietal cortex processes sensory information as well as processing of language and mathematics. As IUGR neonates are shown to exhibit poor neurosensory development, increased rates of language impairments and lower IQ on the mathematics scale [43, 44], this may represent vulnerability of the parietal cortex. Cerebral blood perfusion is not uniform in the IUGR brain and redirection from regions such as the frontal lobe towards the basal ganglia occurs over time [45]. This redistribution may lead to a disturbance in regional brain development that may account for certain disabilities in the IUGR infant. Future studies in pre-clinical animal models are warranted to explore whether certain regions of the brain are selectively susceptible to neuronal and white matter disruption and accordingly inflammatory events.

### White matter disruption in the IUGR piglet brain

White matter disruption is reported to be a major neuropathological feature of the IUGR brain. In this and previous studies, we have demonstrated white matter disruption in the IUGR piglet brain at several fetal time points and postnatally [11]. Our data suggests that even when the fetus has been removed from the compromised in utero environment, disruption to white matter development appears to be ongoing. Like the human brain, the piglet brain is already undergoing myelination [46]. It is well-established from preterm studies that certain oligodendrocyte populations are vulnerable to damage. Some linages may be more vulnerable than others to IUGR conditions which would affect the structure and function of myelination in the IUGR brain. Further characterisation of myelin/oligodendrocyte disruption in the piglet model may be beneficial to distinguish damage to specific oligodendrocyte populations. Determining the mechanisms behind this white matter disruption will aid in the development of treatments to reduce or prevent this ongoing damage.

### Neuroinflammation in the IUGR piglet brain

Neuroinflammation is prevalent in the neonatal hypoxic-ischemic brain and regarded as a key mechanism in the progression of brain injury [47–49]. The IUGR piglet brain also demonstrates evidence of a neuroinflammatory response with increased astrocyte density, increased numbers of activated microglia and elevated proinflammatory cytokines with concomitant decreased anti-inflammatory cytokines. These results are consistent with small animal studies demonstrating an inflammatory response in the IUGR brain with increased microglial activation and astrogliosis [18, 19, 21, 22, 25]. Activation of microglia and reactive astrocytes can produce excessive levels of proinflammatory cytokines such as TNF-α and IL-1β that are toxic to neurons and white matter [50, 51]. However, activated microglia can exist in differing forms: M1 (classic activation; toxic) and M2 (alternative activation; neuroprotective). Depending on the type of insult and the evolution of damage, microglial phenotype can switch over time between M1 and M2 [52–54]. To date, no studies have examined microglial phenotypes in the IUGR brain. Distinguishing the microglial phenotype would be beneficial to determine the ratio of M1 to M2 microglia and therefore the ability to therapeutically exploit these cell types to protect the IUGR neonatal brain.

In this study, we show that neurons are also significant sources of these proinflammatory cytokines in P4 IUGR brains. We demonstrated an increase in neuronal IL-1β in the IUGR brain with no detectable expression on microglia or astrocytes in the parietal cortex. In a rodent model of neonatal encephalopathy, neurons are the first cells to produce excessive levels of IL-1β, earlier than microglia and astrocytes [55]. Savard et al. further work shows IL-1β plays a major role in neuronal self-injury through matrix metalloproteinase (MMP)-9 [56]. We observed IL-1β co-localised with not only neurons but also astrocytes in the PVWM, indicating a potential vulnerable region. Neuronally expressed TNF-α in rodent brain results in microglial activation and an up-regulation of downstream target molecules that leads to peripheral cell infiltration and exacerbation of the inflammatory state [57]. We demonstrate TNF-α not only on neurons but also on astrocytes in the IUGR piglet brain, which may indicate an exacerbated inflammatory state. TNF-α has been shown to induce IL-6 production in astrocytes [58]; we demonstrated a sustained increase in IL-6 gene expression in the IUGR brain supporting this heightened response. We also observed high expression of NF-κB in both microglia and neurons. NF-κB can induce activation of microglia into an inflammatory state that is also neurotoxic [59]. The close interaction we observed between NF-κB-positive microglia and NF-κB-positive neurons may implicate a neurotoxic state in the brain where the microglial processes appeared to wrap around the neuronal cells. The exact mechanisms by which these inflammatory cells induce cellular injury are still not known. However, it is evident that IUGR brains up to P4 display elevated expression of proinflammatory cytokines which is likely contributing to ongoing injury in the brain.

Our findings of increased astrocytic density and alteration in astrocyte morphology are also consistent with IUGR animal studies in sheep and rats [10, 21, 22]. Astrocytes are not only involved in inflammatory events in the brain but are involved in the support and maintenance of neurons, as well as playing a significant role in blood-brain barrier (BBB) dynamics. The BBB is critical to protecting the neonatal brain from toxic substances and maintaining the homeostatic environment. Inflammation, as well as damage to astrocytic endfeet which ensheath the BBB may contribute to BBB compromise in the IUGR brain [60]. In the developing brain, proinflammatory cytokines, TNF-α and IL-1β, can activate MMPs, which accelerate the disruption of the BBB [61]. Microglia are capable of indirectly modulating BBB permeability, by releasing a variety of cytotoxic agents including cytokines, MMPs, nitric oxide and reactive oxygen species [62]. Our finding of astrogliosis, increased microglia and up-regulation of proinflammatory cytokines (TNF-α, IL-1β and IL-6) in the IUGR brain may contribute to BBB damage as recently demonstrated in the sheep IUGR model [60, 63]. Further studies to characterise the extent of BBB disruption in the IUGR piglet brain are warranted.

## Conclusion

While damage to the IUGR fetal brain is known to occur during gestation, it is thought that with removal of the fetus from the compromised environment—birth—damage will cease. Contrary to this, our study demonstrates disruption and damage to brain development continues for several days after birth. We found ongoing neuronal and white matter impairment in the newborn IUGR piglet brain with cellular degeneration and death. This disruption/damage was associated with a neuroinflammatory response with increases in activated microglia, astrocyte density and increased levels of proinflammatory cytokines suggestive of a key role of inflammation in the progression of neuronal and white matter impairment in the IUGR brain. Confirming these mechanisms of injury will help inform suitable postnatal treatment options to reduce brain injury in the IUGR newborn.

## Abbreviations

CXCL10: C-X-C chemokine motif 10
DAPI: 4′,6-Diamidino-2-phenylindole
FJC: Fluoro Jade C
GAPDH: Glyceraldehyde 3-phosphate dehydrogenase
GFAP: Glial fibrillary astrocytic protein
HI: Hypoxic-ischemic
Iba-1: Ionised calcium-binding adaptor molecule-1
IGWM: Intragyral white matter
IL: Interleukin
IUGR: Intrauterine growth restriction
LFB: Luxol Fast Blue
MMP: Matrix metalloproteinase
NF-κB: Nuclear factor-kappa beta
NG: Normally grown
P: Postnatal day
PBS: Phosphate-buffered saline
PCR: Polymerase chain reaction
PVWM: Periventricular white matter
RT: Room temperature
SCWM: Subcortical white matter
TNF: Tumour necrosis factor
